# Medical Reform in France—Convocation General, of Physicians and Surgeons for the Purpose of Devising a Plan of Medical Education and Police

**Published:** 1829-04-01

**Authors:** 


### MEDICAL JURISPRUDENCE.

### XXXIX.

### MEDICAL REFORM.

While the cause of medical reform has been nearly ruined in this country, and all rational reformers disgusted and deterred by the conduct and character of certain brawling demagogues, our Gallic brethren are pursuing a wiser course, and are already almost certain of success. It appears that many petitions on the subject of MEDICAL REFORM, had reached the legislature and been referred to the ROYAL ACADEMY OF MEDICINE; and that a General CONVOCATION of medical men has been called in consequence. This "ASSEMBLÉE GENERALE DES MEDECINS DE PARIS," took place in the "HOTEL du DEPARTEMENT de la SEINE," on the 17th December last, when there were present *one thousand and ninety three* physicians, surgeons, and "praticiens assimilés aux Docteurs," residing in Paris. The president was M. Desgenettes, the vice president was M. Bally, and the secretary was M. Gendrin. This convocation of all regular practitioners, without exception, was for the purpose of electing fifteen of the most talented and distinguished among them as a COMMITTEE for receiving all communications and suggestions—and for drawing up a memorial to government, on the important subjects of medical education, practice, and *police*. The committee includes Desgenettes, Broussais, Rostan, Husson, Magendie, Louyer-Villermay, Bally, Bouillaud, and many others of distinction. M. Desgenettes, after alluding to the message of the Minister of the Interior to the Royal Academy of Medicine, observes that, "however enlightened this body may be, yet the physicians and other medical practitioners of Paris and the Provinces, should have a voice in the proposed ameliorations." "The object was no less than that of fixing legally the basis of medical education and the conditions of qualification—to erect tribunals of discipline, and a medical *police*." "*Il s'agit de fixer légalement les bases de l'enseignement et les conditions des receptions; enfin, de Créer des Chambres de discipline et une police médicale.*" "We believe," says the address, "that all medical practitioners, equal in rights, as they are, in the eyes of the public, whose confidence they possess, ought to be equally consulted on such an important occasion." "Let us confess, gentlemen," says M. Desgenettes, "that our profession stands in need of speedy reform—for the noblest of arts is, with too many, become the vilest of trades." "Car le plus noble des arts, est, entre les mains de plusieurs, le plus vil des métiers."

In the report which the secretary read to this immense assemblage, of the preparatory steps which had been taken by the commission appointed for that purpose, he observes, "the healing art will not render to society all the advantages which society has a right to expect, till the exercise of it is regulated by institutions which shall guarantee not only the proper education but the subsequent *probity* of its professors." "Convinced of the necessity of this, the government, whose solicitude has been awakened by the numerous complaints from all parts of the kingdom, wishes to concentrate all the documents which may tend to form the basis of a law destined to put an end to the innumerable abuses which have crept into the practice of medicine." The report goes on to state that the existing corporate bodies of France have not been able to effect the most salutary regulations, and that if the investigation be confided solely to these bodies, the evils will never be remedied. The following passage we shall not translate:—it is couched in very strong language.

"La nécessité de réformer, si généralement sentie, ne les entraînera-t-elle pas à conseiller des mesures qui pourraient, dans leur application, restreindre des droits acquis, mettre en péril l'indépendance de la profession, l'égalité de tous les hommes qui ont des droits égaux? corps privilégiés eux-mêmes, ne se laisseront-ils pas entraîner à donner leur assentiment à l'établissement de privilèges et de corporations qui, créés sous le prétexte du bien général, au profit de quelques uns, peseraient sur le plus grand nombre?"

It is hardly necessary to remark, that the example which is thus set in Paris, must ultimately be followed in London; for the profession, in this country, is in a still more distracted and wretched state than in France. The idea of establishing a MEDICAL POLICE—that is, a body to watch over and take cognizance of the moral delinquencies, or, more technically speaking, the "breaches of medical ethics," is an excellent one, and such an establishment, we are convinced, will be of the utmost advantage to the profession. We shall watch the proceedings of the committee, and report, from time to time, the results of their deliberations.

